# Signaling from Within: Endocytic Trafficking of the Robo Receptor Is Required for Midline Axon Repulsion

**DOI:** 10.1371/journal.pgen.1005441

**Published:** 2015-09-03

**Authors:** Frédéric Charron

**Affiliations:** 1 Molecular Biology of Neural Development, Institut de Recherches Cliniques de Montréal (IRCM), Montreal, Quebec, Canada; 2 Department of Medicine, University of Montreal, Montreal, Quebec, Canada; 3 Integrated Program in Neuroscience, Division of Experimental Medicine, Department of Medicine, Department of Anatomy and Cell Biology, Department of Biology, McGill University, Quebec, Canada; Max-Planck Institute of Neurobiology, GERMANY

**Fig 1:**
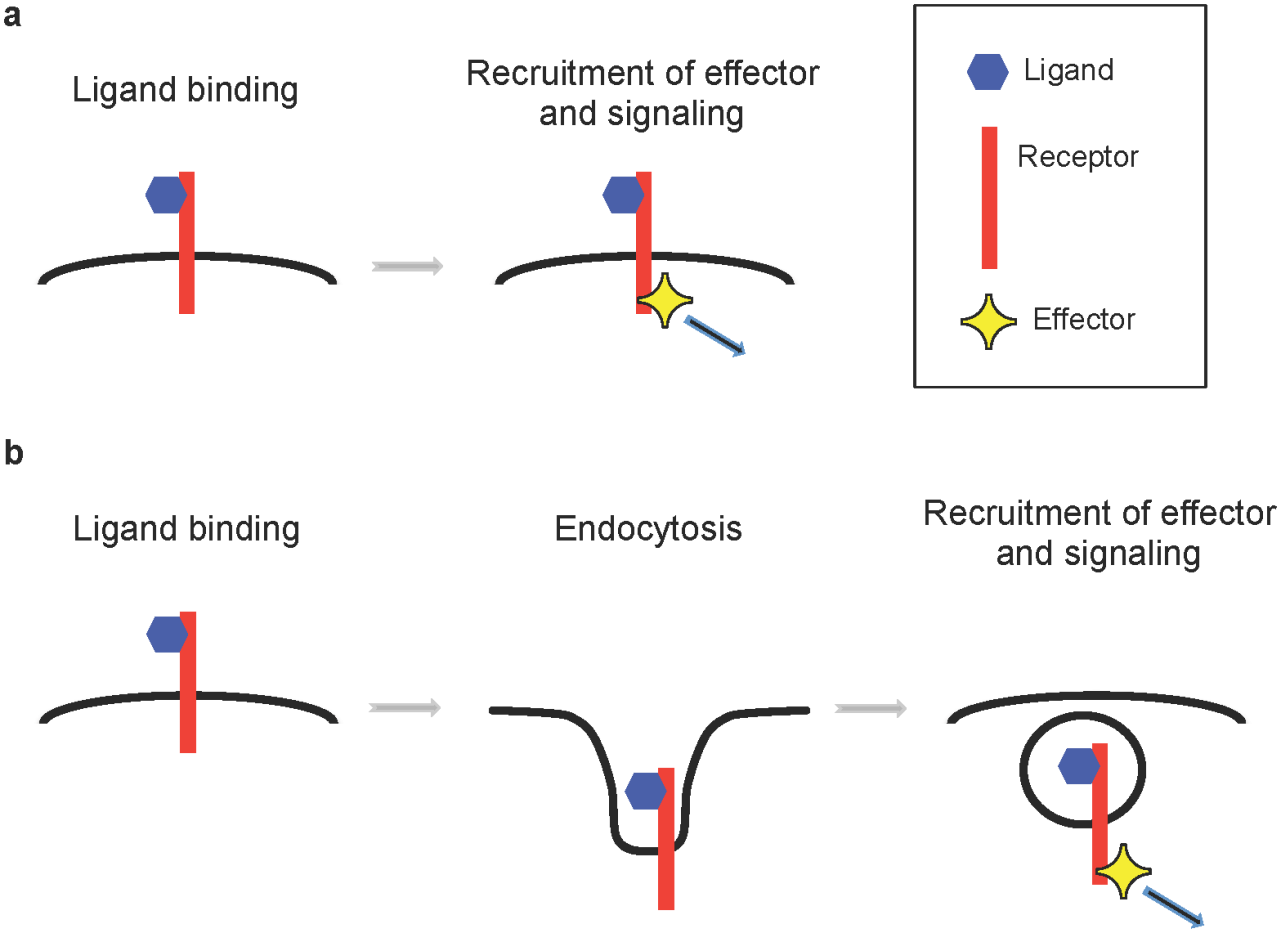
Two models for activation of axon guidance signaling. a) In a first model, the ligand binds to its receptor, causing the recruitment of its downstream effector and signaling at the plasma membrane near the cell surface. b) In a second model, the ligand binds to its receptor, causing the endocytosis of the receptor in endosomes, which leads to the recruitment of its downstream effector and signaling from the endosomes. In an article by Chance and Bashaw, published in this issue of *PLOS Genetics*, they show that Slit binds to its receptor Robo, causing its Clathrin-dependent endocytosis in endosomes and its subsequent recruitment of the effector Sos.

Proper guidance and wiring of axons during development is essential for the normal function of the nervous system. Despite the identification, over the last 20 years or so, of many axon guidance cues and their cognate receptors, we still do not fully understand how their intracellular signal transduction is elicited. Current models often depict that ligand binding to its receptor at the surface of the growth cone leads to the activation of the receptor, recruitment, and/or activation of signaling molecules near the cytoplasmic tail of the receptor, resulting in local signaling emanating from the subcellular compartment where the receptor was located upon binding of its ligand (Fig 1a). An article by Chance and Bashaw, published in this issue of *PLOS Genetics*, uses complementary in vivo and in vitro experiments to challenge this view and show that endocytosis of the Roundabout (Robo) receptor is a key component of receptor activation and precedes the recruitment of its effectors to the receptor cytoplasmic domain [1].

In both invertebrates and vertebrates, Robo receptors comprise a family of axon guidance receptors that mediate repulsion in response to their Slit ligands. In the *Drosophila* embryonic ventral nerve cord, Slit is expressed by midline glia, which creates a repulsive zone for growth cones expressing Robo at their surface. In *robo* mutants, axons that normally do not cross the midline now ignore the presence of Slits and cross or re-cross the midline—as in a roundabout, hence giving the name to the Robo family of receptors.

Endocytosis of axon guidance receptors has previously been shown to modulate axon guidance receptor activity and signaling. For example, endocytic trafficking of Robo by Commissureless prevents its delivery to the growth cone surface. In addition, ligand-dependent receptor endocytosis can be an essential component of growth cone desensitization to guidance cues [2]. This phenomenon may enable growth cones to rapidly attenuate their responsiveness, forming part of an adaptation response that enables growth cones to continually adjust their sensitivity to environmental cues.

In this study, Chance and Bashaw first use *Drosophila* genetics to demonstrate an interaction between Slit-Robo signaling and endocytic pathway components. Specifically, they show that mutants for genes involved in Clathrin-dependent endocytosis (alpha-Adaptin and EndophilinA), or for genes that regulate entry into the early (Rab5) or late (Rab7) endosome, enhance Slit-Robo signaling defects. These results are consistent with endocytosis contributing to Robo activation, as opposed to a change of Robo surface levels available to bind Slit. Interestingly, these endocytic pathway components do not appear to genetically interact with the Netrin receptor Frazzled/DCC, indicating specificity.

To investigate the molecular mechanism underlying the endocytosis of Robo and, importantly, whether Robo is itself endocytosed in a Clathrin-dependent manner, they next turned to an in vitro assay using *Drosophila* cells. Upon Slit ligand stimulation, these cells rapidly undergo spreading of numerous processes in a Robo-dependent manner. In parallel to these morphological changes, Robo is rapidly internalized from the plasma membrane to Rab5-positive early endosomes. Preventing this Slit-dependent trafficking of Robo by either manipulating the endocytic trafficking machinery (pharmacologically or using dominant negative Dynamin and Rab proteins) or mutating conserved AP-2 Clathrin adapter binding sites in the Robo receptor cytoplasmic domain blocks Slit-dependent changes in cell morphology.

These results are consistent with a role for Clathrin-dependent endocytosis in Robo’s ability to elicit signaling, but what effector protein mediates this signaling? The Ras/Rho guanine-nucleotide exchange factor (GEF) Son of Sevenless (Sos) was previously shown to be recruited to Robo in response to Slit stimulation in vitro and to be required for Robo-mediated midline repulsion in vivo [3]. Using their new in vitro assay, the authors show that Slit-dependent endocytosis of Robo is required for its recruitment of Sos. Thus, these results are consistent with a model in which Sos recruitment depends on, and therefore occurs following, Clathrin-dependent endocytosis of the Robo receptor in response to Slit-binding.

The next important step was to test whether Robo endocytosis is important for its activation in vivo. To assay this, the authors used two genetic assays. First, using a gain-of-function assay, they show that Robo mutants that lack AP-2 binding motifs are unable to induce ectopic repulsion from the midline. Second, testing the ability of Robo transgenes to rescue the loss of repulsion defects in *robo* mutant embryos, they show that Robo mutants missing their AP-2 binding motifs are unable to rescue the midline crossing defects of *robo* mutant embryos.

Together, these data strongly support the model that Clathrin-dependent endocytosis of Robo in response to Slit is a critical step in transmitting Robo’s repulsive signal across the plasma membrane. It is remarkable that their data indicates that rather than acting to modulate responses by regulating surface levels, endocytosis of Robo is a key component of receptor activation and precedes the recruitment of Sos to the receptor cytoplasmic domain (Fig 1b). Testing whether Sos is a downstream effector of Robo repulsive signaling in vivo following Robo endocytosis will be a challenging experiment to perform. Nonetheless, these types of experiments could also be useful in the future to reveal the precise spatiotemporal dynamics of cytoskeleton remodeling in a growth cone following Slit binding.

In addition to Sos, Robo also has other downstream effectors, such as Enabled, Dock, and Pak. Future studies will be required to determine if these signaling molecules are also recruited to Robo in an endocytosis-dependent manner or not, and from which subcellular compartment they elicit their signaling activity.

*Drosophila* also has two additional family members, Robo2 and Robo3. It will be interesting to determine whether Robo2 and Robo3 also require endocytosis for their signaling activity. The use of different intracellular and/or endocytic compartments for signaling might impart specificity to distinct family members and thus contribute to fine-tune the development of the nervous system.

Similarly, in vertebrates, four Robo receptors have been identified: Robo1, Robo2, Robo3/Rig-1, and Robo4/Magic Roundabout. The two endocytic motifs found in *Drosophila* Robo are partially conserved in human ROBO1, raising the possibility that endocytosis might also be an important step in vertebrate Robo signaling. Consistent with this idea, Clathrin-dependent endocytosis was found to be required for Slit-induced growth cone collapse of *Xenopus* retinal ganglion cells [4].

Mutations in human *ROBO3* cause horizontal gaze palsy with progressive scoliosis (HGPPS) [5]. Additionally, genetic variations in human *ROBO* receptors have been associated with dyslexia, autism, and schizophrenia [6–8]. It will be interesting to determine whether defective ROBO receptor endocytosis contributes to these pathologies.
